# Liquid Metal as Energy Conversion Sensitizers: Materials and Applications

**DOI:** 10.1002/advs.202304777

**Published:** 2024-03-11

**Authors:** Dawei Wang, Yi Hou, Jianbo Tang, Jing Liu, Wei Rao

**Affiliations:** ^1^ Key laboratory of Plant Resource Conservation and Germplasm Innovation in Mountainous Region (Ministry of Education) School of Pharmaceutical Sciences Guizhou University Guiyang Guizhou Province 550025 China; ^2^ Key Laboratory of Cryogenic Science and Technology Beijing Key Lab of CryoBiomedical Engineering and Key Lab of Cryogenics Technical Institute of Physics and Chemistry Chinese Academy of Sciences Beijing 100190 China; ^3^ School of Future Technology University of Chinese Academy of Sciences Beijing 100049 China; ^4^ School of Chemical Engineering University of New South Wales (UNSW) Kensington NSW 2052 Australia; ^5^ Liquid Metal and Cryogenic Biomedical Research Center Beijing Key Lab of CryoBiomedical Engineering and Key Lab of Cryogenics Technical Institute of Physics and Chemistry Chinese Academy of Sciences Beijing 100190 China; ^6^ School of Future Technology University of Chinese Academy of Sciences Beijing 100049 China; ^7^ Department of Biomedical Engineering School of Medicine Tsinghua University Beijing 100084 China

**Keywords:** energy conversion, liquid metal, sensitizers

## Abstract

Energy can exist in nature in a wide range of forms. Energy conversion refers to the process in which energy is converted from one form to another, and this process will be greatly enhanced by energy conversion sensitizers. Recently, an emerging class of new materials, namely liquid metals (LMs), shows excellent prospects as highly versatile materials. Notably, in terms of energy delivery and conversion, LMs functional materials are chemical responsive, heat‐responsive, photo‐responsive, magnetic‐responsive, microwave‐responsive, and medical imaging responsive. All these intrinsic virtues enabled promising applications in energy conversion, which means LMs can act as energy sensitizers for enhancing energy conversion and transport. Herein, first the unique properties of the light, heat, magnetic and microwave converting capacity of gallium‐based LMs materials are summarized. Then platforms and applications of LM‐based energy conversion sensitizers are highlighted. Finally, some of the potential applications and opportunities of LMs are prospected as energy conversion sensitizers in the future, as well as unresolved challenges. Collectively, it is believed that this review provides a clear perspective for LMs mediated energy conversion, and this topic will help deepen knowledge of the physical chemistry properties of LMs functional materials.

## Graphical abstract

Liquid Metals (LMs) featuring both fluidic and metallic properties, are capable of responding to external energy stimuli, which are particularly suitable for energy conversion in many impactful applications. This review systematically summarized and discussed representative platforms and applications of the LMs‐based energy conversion sensitizers, with an emphasis on the intrinsic energy‐responsive virtues and the involved mechanism for specific energy conversion.

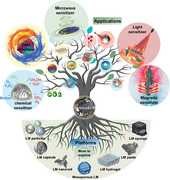

## 1 Introduction

Energy is everywhere in human life, it may exist in electric energy, thermal energy, light energy, chemical energy, mechanical energy and other various forms.^[^ ^1^ ^]^ The energy with different forms can be converted to each other through physical effects or chemical reactions, and the transferring or conversion of these energies often requires the transmission of energy sensitizers (or energy‐responsive materials).^[^ ^2^ ^]^ For example, in the field of heat dissipation, the traditional way of heat dissipation is to enhance heat exchange through heat transfer by air. If high thermal conductivity materials are applied instead of air, the energy transfer efficiency would be greatly enhanced. In the field of photothermal conversion, materials with high photothermal conversion efficiency would improve the efficiency of photothermal conversion.^[^ ^3^ ^]^ Improving the efficiency of energy transfer or conversion is another level of energy conservation. One of the great challenges in the twenty‐first century is unquestionably the energy conversion concerns. Therefore, the investigation of new energy sensitizers holds one of the keys to improving energy conversion efficiency.

In recent years, room temperature liquid metals (LMs for short) have been gradually approaching researchers’ vision and have shown excellent prospects as highly versatile materials. The natural physicochemical properties of LMs show both characteristics of fluid and metal, which have been continuously disclosed and subsequently leveraged. This revolutionary discovery has changed our inherent understanding of metals and has had a huge impact in a wide range of areas such as soft electronics, composites, catalysis, and biomedicine.^[^ ^4^, ^5^ ^]^ Furthermore, gallium (Ga)‐based LMs can be functionalized to a wide range of shapes and sizes.^[^ ^6^ ^]^ When compared to mercury, the low toxicity of Ga opens up a broader field, particularly in biomedical fields.

Particularly, a series of unique characteristics of LMs have been found very recently, including chemical response, heat response, laser response, magnetic response, microwave response, and medical imaging response. Within a specific application, these intrinsic energy‐responsive virtues are beneficial for energy conversion (conversion of energy into another form, e.g., chemical energy converting into kinetic energy, light energy converting into heat energy, etc.) accompanied by changes in physical and chemical properties, which means LMs could act as energy sensitizers for enhancing energy conversion or transport. First, the LMs possess strong electronegativity and favorable electrochemical potential window, which offer potential chemically induced responses for energy conversion, such as energy capture and storage (e.g., catalysis for fuel generation), and self‐driven motors (converting chemical energy into mechanical actuation).^[^ ^5^, ^7^ ^]^ While, the heat response means the superior high thermal conductivity of LMs, which makes them potential as convective coolants and thermal interface materials. In 2002, Ga‐based LMs were first proposed as a coolant for the cooling of high‐performance chips, after that LMs materials mediated thermal management technologies have been widely developed.^[^ ^8^ ^]^ Besides, LMs composites with other high thermal conductivity particles have been investigated and are expected to break through the cooling limit of the conventional cooling method (air and water cooling). Then, in 2016, the optical property of LMs was studied and it was found that LMs nanocapsules could generate heat and reactive oxygen species (ROS) under near‐infrared (NIR) laser irradiation.^[^ ^9^ ^]^ After that, researchers synthesized a wide range of LMs functional materials and confirmed their photothermal performance, shape transformation, ROS generation, and enhanced photoacoustic imaging under NIR laser irradiation.^[^ ^9^, ^10^, ^11^, ^12^, ^13^ ^]^ Studies demonstrated that not only LMs nanoparticles (NPs), but also LMs paste, and LMs hydrogel (ranging from nano‐size to macro size) all show excellent photothermal conversion performance. Therefore, Ga‐based LMs materials with photothermal effects have been applied in tumor ablation under NIR‐triggered therapeutics. Besides optical properties, the electromagnetic response of LMs has also been investigated.^[^ ^14^, ^15^ ^]^ Interestingly, under an alternative magnetic field (AMF), LMs could generate inductive heating and show excellent magnetic heating performance when compared with conventional magnetite materials (eg, Fe_3_O_4_). The intrinsic virtue of this property enabled promising application in hyperthermia therapy under AMF.^[^ ^16^, ^17^ ^]^ Moreover, very recently, it was revealed that LMs supernanoparticles could generate ROS under microwave irradiation. This finding is expected to improve the efficacy of the dynamic treatment for deep‐seated tumor therapy in clinical studies.^[^ ^18^ ^]^

**Figure 1:**
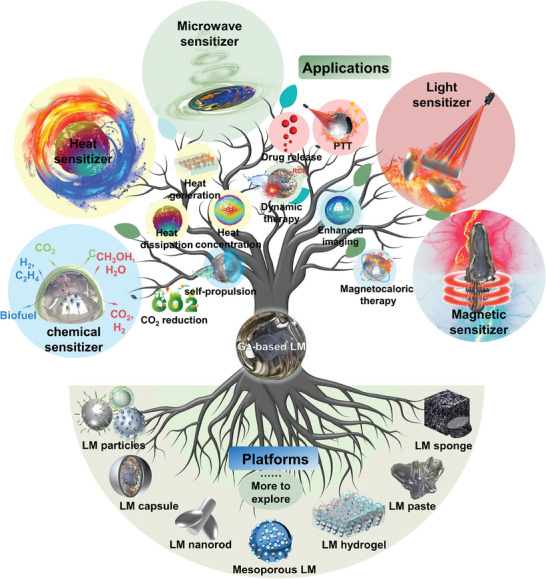
Platforms and applications of LMs as energy conversion sensitizers.

Up to now, reviews about LMs in chemistry, biomedical applications, composites, robots, thermal management, and functionalization have emerged tremendously. However, no report focuses on the energy conversion perspective. This review seeks to summarize and highlight the platforms and applications of LMs as energy conversion sensitizers, including chemical sensitizers, heat sensitizers, light sensitizers, magnetic sensitizers, microwave sensitizers, and imaging sensitizers (**Figure** **1**). We first focus on the unique properties of LMs covering energy conversion, including chemical response, heat response, light response, magnetic response, and microwave response. In this part, we also summarized different LMs materials that possess these unique properties and tried to explain the involved mechanism of these properties. Then platforms and applications of different LMs types in achieving enhanced energy conversion or transport will be presented. Finally, this review also offers some of the potential applications and opportunities of LMs as energy conversion sensitizers in the future, as well as unresolved challenges.

## 2 Characteristics of LMs Covering Energy Conversion

Liquid metals (LMs) are usually defined as metals or alloys with low‐melting points below or near room temperature. The two most commonly used Ga‐based LMs are eutectic gallium indium (EGaIn, 75% Ga and 25% In by weight) and gallium indium‐tin (typically 68% Ga, 22% In and 10% Sn by weight).^[^ ^19^ ^]^ LMs have different physical and chemical properties depending on their composition and proportion. **Table** **1** shows the typical properties of LMs, air, and typical liquids. It is obvious that LMs share the properties of both metals and fluids. Pure Ga and EGaIn have the melting point of 29.8 °C and 15.7 °C, which keep them in a liquid state easily at room temperature. Although LMs show slightly lower electrical and thermal conductivity than molten Tin, they show much higher thermal conductivity, high electrical conductivity, good fluidity, and low viscosity when compared with water. And by modifying or doping LMs, new physical and chemical properties can be revealed.

**Table 1:** Properties of LMs, air and typical liquids.

	Air	Water	Mercury	C_10_H_15_F_6_N_3_O_4_S_2_	Tin (white)	Pure Ga	GaIn_24.5_	GaIn_21.5_Sn_10_
Electrical conductivity (S/m)	0	5.5 × 10^−7^	1.04 × 10^6^	0.433	8.7 × 10^6^	≈3.8 × 10^6^	≈3.4 × 10^6^	3.3−3.5 × 10^6^
Thermal conductivity (W/(m·k)	0.023	0.599	8.51	0.128	33.72^a)^	28.4−30.5^b)^	25.9−26.4^b)^	24.9–25.4^b)^
Heat capacity (J/(kg·k)	1.003	4183	140	–	257.43^a)^	397.6	403.5	296–392^b)^
Density (g/cm^3^)	1.293 × 10^−3^	1.0	13.55	1.457	6.94^a)^	6.09	6.28	≈6.4
Melting point (°C)	−218.79	0	−38.8	11.0	231.9	29.8	≈15.5	≈10.0
Viscosity (10^−6^ m^2^/S)	14.8	1.002	0.114	61.8	≈0.22^a)^	≈0.324	≈0.40	≈0.32
Magnetic susceptibility (10^−6^cm^3^mol^−1^)	–	−12.96	–	–	–	−21.6	–	–
Ref.	[20]	[20, 21]	[20]	[20]	[22]	[20, 23, 24]	[15, 25, 26]	[24, 25, 27]

Note: C10H15F6N3O4S2: 1,2‐Dimethyl‐3‐propylimidazolium bis(trifluoromethylsulfonyl)imide;

^a)^ at 300 °C; Others around room temperature;

^b)^ The physical properties of LMs are affected by the test method, temperature, environment, and oxide layer.^[^ ^24^, ^28^ ^]^

Recently, besides the properties mentioned in Table 1, some unique characteristics of LMs have been found, including heat response, light response, magnetic response, microwave response, and imaging response. These unique properties make LMs potential as energy conversion sensitizers. The high thermal conductivity endows LMs superior advantage in thermal management, especially as a coolant and thermal interface material, which means LMs here play the role of heat sensitizers. Due to the plasmonic effect, LMs show an excellent photothermal conversion effect, which makes it possible to be a light sensitizer in photothermal therapy. Besides light response, LMs have been proved to be microwave response and magnetic response during microwave irradiation and AMF. We will elaborate on these unique properties of LMs and the mechanism for their possession.

### 2.1 Fluidic Property

**Figure 2:**
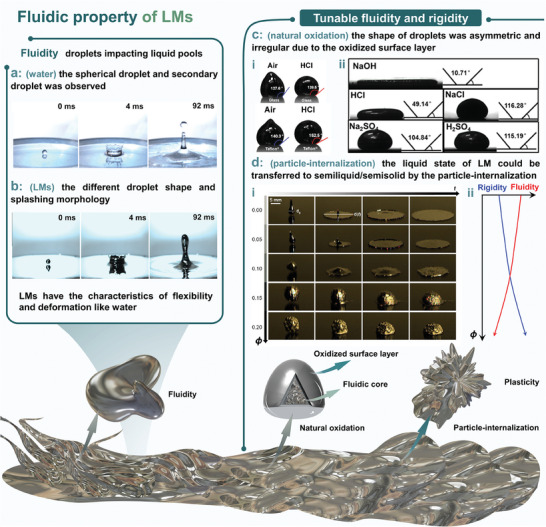
The fluidic property of LMs. Comparison of a) water and b) GaIn_24.5_ droplets impacting their respective liquid pools during the splashing processes at the same moment. Reproduced with permission.^[^ ^30^ ^]^ Copyright 2014, Elsevier. c‐i) Contact angle of a Galinstan droplet on a bare glass slide and a Teflon‐coated glass slide in air and after exposure of the droplet to HCl vapor. Reproduced with permission.^[^ ^31^ ^]^ Copyright 2013, American Chemical Society. ii) Contact angle of the LMs with the graphite substrate in different electrolyte solutions. Reproduced with permission.^[^ ^32^ ^]^ Copyright 2018, American Chemical Society.d). i) The impacting tests LM‐Cu samples reveal liquid‐ to the solid‐like transition of their impacting behaviors as ϕ increases. ii) Schematic demonstration of fluidity decreases and rigidity increases of the LM‐Cu samples as ϕ increases. Reproduced with permission.^[^ ^33^ ^]^ Copyright 2017, American Chemical Society.

The most significant difference between LMs and other solid metals is their unique fluidity, distinguishing them from solid metals for a more flexible and efficient platform. The fundamental reason for LMs maintaining fluidity is their low melting point and high boiling point, thus enabling them to behave as liquids over an extremely wide range of temperatures. It is worth mentioning that the melting point and boiling point of Ga‐based alloys could be adjusted according to their elemental composition and proportion. For example, the melting point of pure Ga is 29.8 °C, while that of Ga‐based alloys could be adjusted to a lower temperature (e.g., 15.7 °C for GaIn_24.5_ and −19.0 °C for GaIn_21.5_Sn_10_). The boiling point of pure Ga is 2204.8 °C, while that of Ga‐based alloys is markedly decreased (e.g., ≈2000 °C for GaIn_24.5_ and ≈1300 °C for GaIn_21.5_Sn_10_). Because of its fluidity, LMs have the characteristics of flexibility and deformation like water (**Figure** **2**a,b). Nevertheless, due to the oxidation effect of LMs, the droplet shape and the splashing morphology of LMs were drastically different from those of water. As shown in Figure 2a,b, a spherical droplet and a second droplet of water are observed, while the secondary droplet is hard to form of LMs as a result of higher dynamic viscosity and surface oxidation.^[^ ^29^ ^]^ Due to the spontaneous formation of a solid oxide surface layer, the LMs droplets possess different interfacial properties, such as surface tension, wettability, and mechanical strength. Figure 2c‐i shows the droplets of naturally oxidized LMs on two different types of substrates (glass and Teflon‐coated glass) before and after HCl vapor treatment. We can see that the shape of the droplets was asymmetric and irregular in the air, while the contact angle changed rapidly after HCl vapor evaporated and reacted with the oxidized surface layer of the LMs droplet. In addition, contact angles could also be altered when LMs droplets contact with graphite in electrolyte solutions, including NaOH, NaCl, Na_2_SO_4_, HCl, and H_2_SO_4_ (Figure 2c‐ii). More interestingly, the liquid state of LMs could be transferred to semiliquid/semisolid by the particle‐internalization process of LMs and Cu particles (Figure 2di). With the packing ratio (ϕ = weight of Cu particles/ weight of LMs) increases, the appearance of the LMs changes from fluid to paste (fluidity decreases and rigidity increases, Figure 2d‐i), demonstrating the tunable fluidity and rigidity of LMs (Figure 2d‐ii).

### 2.2 Chemical Response

The Ga‐based LMs possess strong electronegativity and favorable electrochemical potential window, which offer potential chemically induced responses for energy conversion, such as energy capture and storage (e.g., catalysis for fuel generation), and self‐driven motors (converting chemical energy into mechanical actuation).^[^ ^5^, ^7^ ^]^

#### 2.2.1 Spontaneous Formation of Oxide Layer

Even in trace amounts of oxygen, an atomic oxide layer (thickness 0.7–3 nm^[^ ^34^, ^35^ ^]^) would spontaneously form at the LMs‐environment interface.^[^ ^36^, ^37^ ^]^ For Ga‐based LMs (e.g., Ga, GaIn, or GaInSn alloys), the oxide formation occurs sequentially due to the difference in reduction potentials (E0:, e.g., Ga (−0.56 V) < In (−0.34 V) < Sn (−0.14 V)), which ultimately dominating the exterior surface composition (mainly consists of Ga–O bilayers).^[^ ^38^ ^]^ This oxide layer is similar to a protective aluminum oxide layer, which acts as a partially passivating barrier against further oxidation.^[^ ^36^ ^]^ It is worth noting that the oxidation of LMs is complex and depends on many factors, such as alloy composition, stoichiometry, oxidant diffusion, microstructure, cohesive energy density, temperature, and pressure.^[^ ^36^ ^]^ For example, the oxidation process and oxidation products of Ga‐based LMs are varied when exposed to different environments, e.g., in the dry air: 4Ga + O_2_ = 2Ga_2_O (unstable), 4Ga + 3O_2_ = 2Ga_2_O_3_; in aqueous solution or humid air: 4Ga + 2H_2_O + 3O_2_ = 4GaOOH, 2Ga + 4H_2_O = 2GaOOH + 3H_2_↑. Meanwhile, the formation of the surface oxide layer can be continuously intensified or inverted in response to external excitations (e.g., chemical, electrochemical or mechanical excitations) and the alteration of reaction conditions (e.g., ingredients, temperature, and time).^[^ ^36^ ^]^ For example, the oxidant addition, increased temperature and oxygen atmosphere exposure time may disrupt the oxide layer and enhance oxygen permeability, thereby intensifying further oxidation. Conversely, due to its amphoteric reactivity, the oxide layer is fragile and can be easily eliminated in the presence of strong acids, bases, or electrolysis.

The oxidation regulation is an effective approach to alter the fundamental physicochemical properties of LMs materials (e.g., composition, morphology, structure, surface adhesion, and rheological properties), and can even confer fascinating functions (e.g., shape transformation, super‐cooling effect, and stimulus responsiveness).^[^ ^28^, ^36^ ^]^ For instance, the oxide layer serves as an essential component, may significantly decrease the surface tension and increase the stickiness and plasticity, which is critical for better adhesion to the substrates (e.g., for LMs‐based electronics prepared via direct ink writing and transfer printing methods).^[^ ^35^ ^]^ Besides, the oxide layer may hinder heterogeneous nucleation and prevent the liquid–solid phase transformation, ultimately leading to a more significant super‐cooling effect.^[^ ^39^ ^]^ In addition, the hyperthermal oxidation may also lead to the shape transformation phenomenon, which is beneficial for intracellular penetration, drug release, and endosomal escape, showing great promise in the biomedical field.^[^ ^9^, ^40^, ^41^ ^]^ However, the oxidation may also bring some negative effects, which is sometimes considered as a nuisance due to the deterioration of stability and performance.^[^ ^28^, ^36^ ^]^ For example, the internal equilibrium of the multicomponent alloying LMs (e.g., ternary LMs, GaInSn) will be disrupted with continuous oxidation, leading to dealloying, localized enrichment, and precipitation of alloying elements.

#### 2.2.2 Electrochemically Induced Energy Conversion

Due to the fluidic nature and chemical reactivity, LMs are able to facilitate the energy conversion from spontaneous chemical/electrochemical reactions into catalytic synthesis (e.g., hydrogen generation, CO_2_ reduction, and methanol synthesis) and mechanical actuation (e.g., chemical propulsion, and electrochemical propulsion).^[^ ^5^, ^42^, ^43^ ^]^

**Figure 3:**
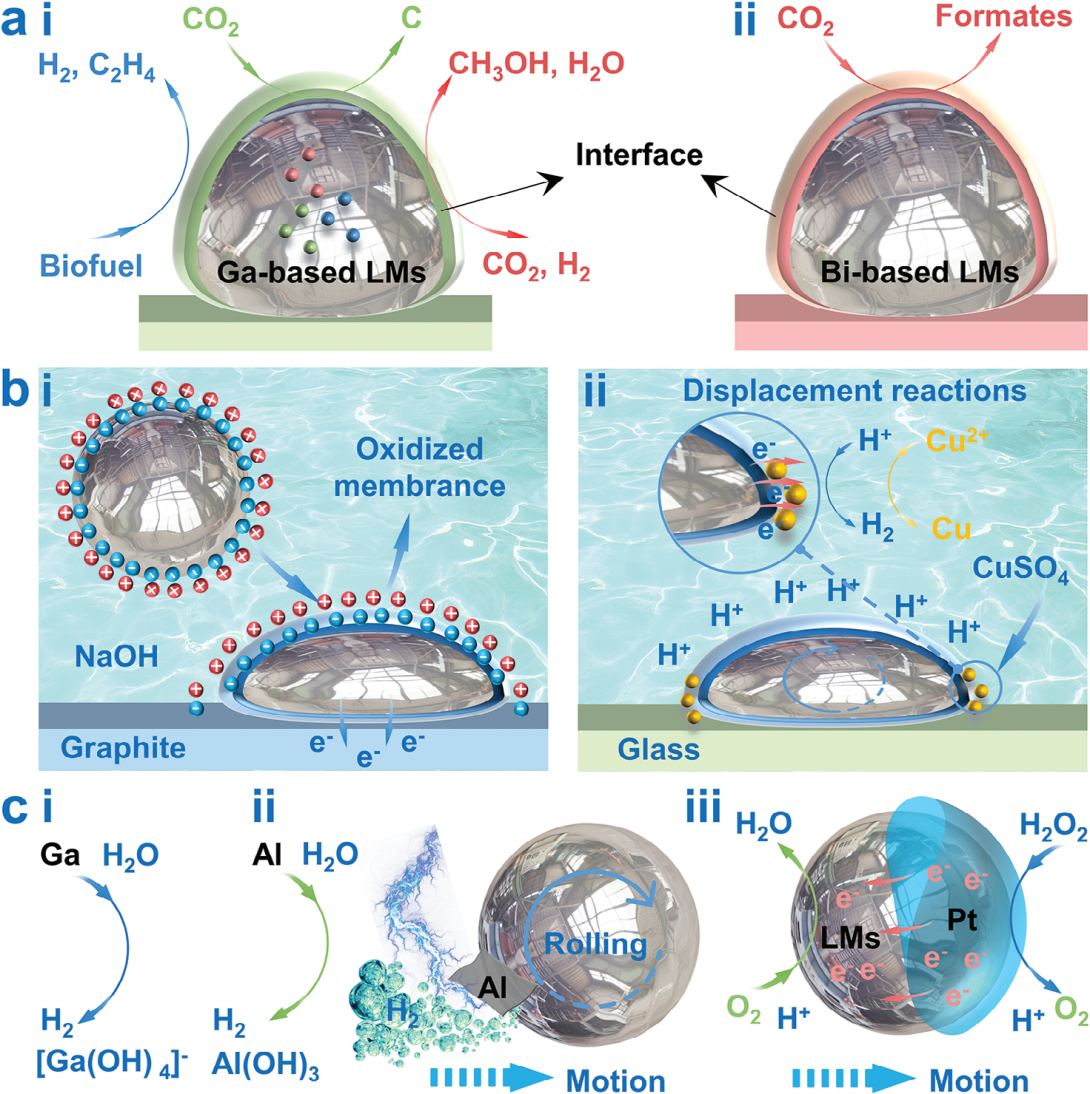
Electrochemically induced energy conversion. a) LMs‐based catalysts for catalytic synthesis; i) Ga‐based LMs containing additional metallic elements in the form of supersaturated nanoparticles, solutes, and intermetallic compounds; ii) Bi‐based LMs. b) The surface tension regulation to trigger the deformation or motion of LMs; i) LMs‐graphite galvanic cell; ii) Cu–Ga galvanic cell. c) The autonomous movement of LM droplets, via i) hydrogen bubbles generation enhanced by electric field or ii) actuating fuel introduction, and iii) self‐electrophoresis.

Nowadays, a series of novel LMs‐based catalysts have been proposed to provide more sustainable catalytic pathways to address current global challenges, such as greenhouse‐gas‐emission conversion, and sustainable energy production (**Figure** **3**a).^[^ ^5^ ^]^ In the molten state, monophasic LMs can accommodate additional trace metallic elements through dissolution,^[^ ^44^ ^]^ thus establishing efficient liquid catalyst systems (Figure 3a‐i).^[^ ^45^, ^46^, ^47^ ^]^ For instance, the pure liquid‐Ga or Galinstan alloy that contains metallic elemental cerium (Ce) nanoparticles (up to 3 wt.%) could serve as electrocatalysts, thereby effectively facilitating the electrochemical reduction of CO_2_ to layered carbonaceous species (solid).^[^ ^47^ ^]^ While the Ce nanoparticles and the cerium oxide catalyst (formed at the LMs/electrolyte interface) are key to jointly promoting the room temperature reduction reaction of CO_2_. Compared to the solid‐state catalysts that are commonly associated with coking and coarsening limitations and short lifetime, the LMs‐based liquid‐phase catalysts exhibit superior coking resistance since the carbonaceous product would spontaneously peel off from the liquid interface (due to the suppressed van der Waals adhesion). Besides, the trace amounts of platinum (Pt) can naturally dissolve in liquid Ga and exist in liquid form without atomic segregation, which can effectively activate surrounding Ga atoms for catalysis.^[^ ^45^ ^]^ At low temperatures (318‐343 K), this liquid catalyst system can actuate a series of catalytic reactions (e.g., electrochemical oxidation of pyrogallol (PG), 3,3′,5,5′‐tetramethylbenzidine (TMB) and methanol (CH_3_OH), and reduction of methylene blue (MB)) with improved kinetics, showing superior catalytic performance than solid Pt catalysts.^[^ ^45^ ^]^ Moreover, the conversion of renewable biofuels into environmentally sustainable energy sources (mostly H_2_ and C_2_H_4_) and the conversion of CO_2_ and H_2_ to value‐added products (e.g., methanol) can also be achieved by employing liquid Ga–nickel (Ni) as co‐catalysts (Ga–Ni catalysts) with mechanical energy excitation.^[^ ^46^, ^48^ ^]^ In addition to Ga‐based LMs, other low‐melting‐point alloys, such as binary (e.g., Sn–Bi alloy) and ternary (e.g., Field's metal, In_51.0_Bi_32.5_Sn_16.5_) alloy LMs, have also shown remarkable catalytic properties (Figure 3a‐ii).^[^ ^49^, ^50^ ^]^ For example, the electroconversion efficiency of CO_2_ toward formates using Sn–Bi alloy LMs or Field's metal has been demonstrated to exceed 90%^[^ ^49^ ^]^ and impressive 82%, respectively.

In addition, the soft LMs microdroplets may undergo significant bending, stretching, deformation, and even autonomous movement in response to chemical action along with other forces (including electrical, and electrochemical stimulants).^[^ ^43^ ^]^ Basically, the redox chemistry on the LMs surface can be activated by constructing galvanic cells within conductive chemical environments, thus providing an effective strategy for regulating the surface tension and triggering the deformation or motion of LMs (Figure 3b). For example, the surface tension gradients can be induced by establishing LMs‐graphite galvanic cell (on a graphite plate, LMs immersed in acidic electrolyte with H_2_O_2_ addition, Figure 3b‐i)^[^ ^51^ ^]^ and Cu–Ga galvanic cell (LMs immersed in CuSO_4_ solution with acidic electrolyte addition, Figure 3b‐ii),^[^ ^52^ ^]^ leading to rapid diffusion and fractal behavior of LMs. In particular, the LMs can also convert chemical energy into mechanical actuation via bubble generation, electric potential difference, and self‐electrophoresis, thus enabling the autonomous movement of LM droplets (Figure 3c).^[^ ^53^, ^54^ ^]^ The metallic element Ga possesses amphoteric chemical properties similar to aluminum (Al), which allows Ga‐based LMs to release hydrogen bubbles in an electrolyte. In order to provide sufficient driving force, the hydrogen bubble generation can be enhanced by external electric field stimulation,^[^ ^55^ ^]^ or actuating fuel introduction.^[^ ^54^ ^]^ For example, the electric field is simultaneously capable of inducing electric potential gradient across the electric double layer (induce pressure difference) and surface tension gradient on the LMs surface, accompanied by the hydrogen bubbles generation, which ultimately generate a strong driving force to actuate the LMs droplets (Figure 3c‐i).^[^ ^55^ ^]^ Except as reactants to generate hydrogen bubbles, LMs can also be used as catalysts to facilitate water splitting. Typically, the Al‐water reaction is considered as an alternative method for hydrogen generation, however, this method is severely limited by the spontaneously formed dense oxide layer that prevents the reaction from proceeding. Fortunately, the LMs may cause a severe disruption of the lattice structure when in contact with solid metals (called LMs embrittlement effect^[^ ^56^ ^]^), which can be utilized for catalyzing Al‐water reaction.^[^ ^53^, ^54^ ^]^ Previous studies have shown that Ga‐based LMs (including pure Ga,^[^ ^57^ ^]^ Ga‐Sn/In/Zn binary alloys,^[^ ^58^, ^59^ ^]^ Galinstan ternary alloys,^[^ ^58^, ^60^ ^]^ and Ga/Bi/Pb/Sn multicomponent alloys^[^ ^61^ ^]^) are all able to significantly enhance Al activity toward the Al‐water reaction through the size reduction (increasing the reaction surface area) of the Al‐reactant, the impeded formation of aluminum oxides and the decreased surface energy.^[^ ^5^ ^]^ Therefore, the LMs droplets, after engulfing a small Al flake as fuel, can realize self‐propulsion through the forces generated by the massive hydrogen bubble, and the bipolar electrochemical reaction induced imbalanced surface tension (Figure 3c‐ii).^[^ ^54^ ^]^ Furthermore, the platinum/Galinstan Janus particles could also self‐propel in H_2_O_2_ solution, due to the self‐electrophoresis mechanism induced by asymmetric protons distribution (Figure 3c‐iii).^[^ ^53^ ^]^

### 2.3 Heat Response

**Figure 4:**
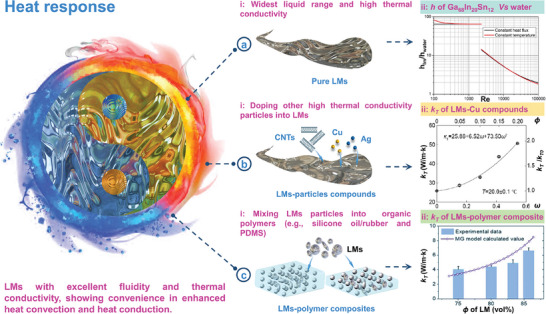
Thermal properties of LMs. a) Pure LMs: i) pure LMs with fluidity and metallicity, showing extremely wide liquid range and high thermal conductivity; ii) the ratio of heat transfer coefficient of water and Ga_68_In_20_Sn_12_ versus Re number. Reproduced with permission.^[^ ^63^ ^]^ Copyright 2014, Elsevier. b) LMs‐particles compounds: i) the thermal conductivity of LMs‐ particles compounds can be greatly improved by doping other high thermal conductivity materials, such as carbon nanotubes, copper particles, and silver particles; ii) thermal conductivity (k_T_) measurement of LM‐Cu composite as a function of the mass ratios of CuGa_2_ (ω) and Cu (ϕ), respectively. Reproduced with permission.^[^ ^33^ ^]^ Copyright 2017, American Chemical Society.c) LMs‐polymer composites: i) LMs can be mixed into organic polymers (such as silicone oil, silicone rubber, and polydimethylsiloxane (PDMS)) to achieve thermally‐conductive LM‐polymer composites; ii) thermal conductivities of the thermally‐conductive LM‐polymer composites showing good consistency with Maxwell‐Garnett's model prediction. Reproduced with permission.^[^ ^68^ ^]^ Copyright 2018, The Royal Society Of Chemistry.

Distinguished from conventional liquids, LMs can be regarded as an amorphous metal without any crystalline lattice (have water‐like viscosity), consisting of positively charged metal ions and metal atoms generated free‐electron cloud.^[^ ^5^, ^62^ ^]^ Since the delocalized electrons of LMs can freely interact with a thermal energy field, it bestows them high thermal conductivity (**Figure** **4**). At room temperature, the thermal conductivity of pure Ga is ≈50 times higher than that of water (29.28 W/(m·k) Vs. 0.599 W/(m·k)), demonstrating its excellent heat transfer capacity. Figure 4a‐ii presents the variation of the heat transfer coefficient ratio (h_LMs_/h_water_) under different Re numbers. In the case of laminar flow conditions, the cooling capability of pure LMs (Ga_68_In_20_Sn_12_) is much superior to that of water; under turbulent flow conditions, this advantage decreases because the performance of water is greatly enhanced due to turbulence.^[^ ^63^ ^]^ In addition, the thermal conductivity of LMs can be greatly improved by doping other discontinuous‐phase materials such as carbon nanotubes, copper particles, and silver particles into LMs (Figure 4b‐i). As shown in the following **Table** **2**, the thermal conductivity of typical LMs‐particles compounds that are doped with carbon nanotube (CNTs)^[^ ^64^ ^]^ and graphene^[^ ^65^ ^]^ can reach ≈67.3 W/(m·k) and 82.8 W/(m·k), which is 2–3 times higher than that before mixing. Besides, the improvement of thermal conductivity is more significant with the increase of the packing ratio (ϕ = weight of doped particles/ weight of LMs). For example, when Cu particles were doped into LMs, the thermal conductivity gradually increased as the packing ratio of Cu (ϕ) and the mass ratios of CuGa_2_ (ω; ω = ϕ (1 + 2M_Ga_/M_Cu_)/(1 + ϕ), where M_Ga_ and M_Cu_ are the molar mass of Ga and Cu, respectively) increase (Figure 4b‐ii).^[^ ^33^ ^]^ This particle doping strategy can not only effectively improve thermal conductivity but also show long‐term stability and durability.^[^ ^33^ ^]^ Moreover, LMs can also be mixed into organic polymers (such as silicone oil, silicone rubber, and polydimethylsiloxane (PDMS)) to achieve thermally‐conductive LMs‐polymer composites (or called LM embedded elastomers (LMEEs)^[^ ^66^, ^67^ ^]^) (Figure 4c‐i). For the LMs‐polymer composites, the thermally‐conductive LMs droplets are dispersed in the matrix and directly in contact with the polymer, which may form a relatively stable and efficient heat transfer path, thus improving the thermal conductivity. While, the thermal conductivity of LMs‐polymer composites may increase with the proportion of the filling LMs droplets, owing to the formation of more thermal conduction paths after more frequent contact among these inclusions (Figure 4c‐ii).^[^ ^68^ ^]^ It is worth noting that using LMs microdroplets as inclusions could develop thermally‐conductive composites, and may become electroconductive under stress (destruction of the surface oxide layer to realize electroconductive pathways, e.g., mechanical stresses,^[^ ^69^ ^]^ thermal stress^[^ ^70^ ^]^ and acoustic shock^[^ ^67^, ^71^ ^]^) or hypothermic stimulation (volume expansion to realize electroconductive pathways^[^ ^72^ ^]^). While using LMs nanodroplets as inclusions, thermally‐conductive and electrically‐insulating composites can be prepared to prevent the risk of electrical leakage or corrosion caused by LMs precipitation. In general, with the rapid development of information technology, especially in the electronics industry, generous heat needs to be dissipated more quickly in a smaller space, so it is of great urgency for researchers to look for better coolants to meet these challenges. In this case, LMs functional materials have been widely used in the field of chip heat dissipation and thermal management due to their excellent thermal transmission and thermal extraction capabilities.

**Table 2:** Thermal properties of typical materials based on LMs.

Property	LMs‐particles composite						LMs‐polymer TIM		
Matrix	EGaIn	Ga	Ga	GaInSn	Ga	Ga	Silicone oil	Silicone elastomer	PDMS
Additive	Cu	Au	Ag	Ni	CNTs	graphene	EGaInSn	EGaIn	GaInSn
Packing ratio	20 wt.%	20 vol%	3 wt.%	–	20 vol%	1.5 wt.%	19.2 vol%	50 vol%	50 vol%
Thermal conductivity (W/(m·k))	50.0	45.9^a)^	46.0	33.3	≈67.3^a)^	82.8	5.3	4.7 ± 0.2	1.5 ± 0.1
Ref	[33]	[64]	[73]	[74]	[64]	[65]	[75]	[76]	[77]

Note:

^a)^ via Theoretical calculation.

### 2.4 Light Response

**Figure 5:**
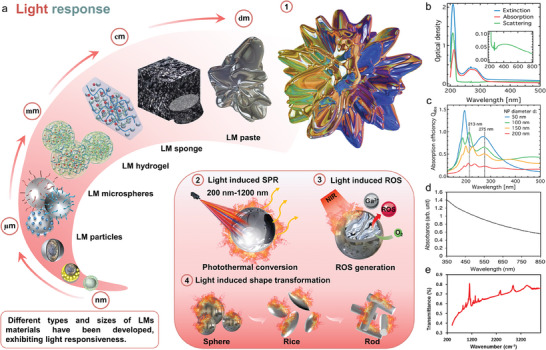
a) The light responsiveness of LMs materials with different types and sizes. 1) colorful luster under the interference of light, 2) light induced surface plasmon resonance (SPR), 3) light induced ROS generation, 4) light induced shape transformation. The optical performance of LMs NPs: b) Extinction, absorption, and scattering spectra of the EGaIn NPs. c) Calculated absorption efficiency Q_abs_ as a function of wavelength for 50, 100, 150, and 200 nm sized particles. Reproduced with permission.^[^ ^81^ ^]^ Copyright 2019, Springer Nature. d) Ultraviolet‐visible–NIR absorbance spectrum of LMs NPs. e) Transmittance spectrum of LMs NPs. Reproduce with permission.^[^ ^82^ ^]^ Copyright 2019, Elsevier.

As a high‐frequency electromagnetic radiation, light offers unique advantages in energy conversion since the light properties (e.g., intensity and frequency) and irradiation parameters (e.g., irradiation direction, position, area, and duration) could be remotely regulated or converted via the light sensitive materials. Although the optical properties of LMs functional materials, unlike mercury and traditional metals, have been widely and deeply studied, their unique optical properties have been gradually discovered very recently. Nowadays, LMs materials with different light responsiveness have been successfully developed through strategies such as size adjustment, structural design, surface modification, and oxidation regulation, as shown in **Figure** **5**a.

Generally speaking, the macroscopic LMs present a silver‐white sheen under natural light, because most of the incident light (electromagnetic energy with different frequencies) is reflected after interacting with the free electrons inside the LMs. While the scales down to microns and nanometers, LMs particles turn black due to the anisotropic reflection between numerous particles.^[^ ^78^ ^]^ For the first time, it was found that macroscopic LMs could be anodized to iridescent color in an electrolyte solution. The mechanism involved is the formation of porous nanoscale oxide film on the surface of the LMs after anodizing, which could be fine‐tuned to control the incident light frequency via light interference, thus forming a colorful luster (Figure 5a‐1).^[^ ^78^ ^]^

In addition to the light properties regulation, LMs with nano‐size can also convert light energy into other forms as a result of the interaction between the light, ambient environment, and LMs‐based light sensitive materials. In 2017, LMs nanoparticles (LMs NPs) were found to produce a lot of heat (Figure 5a‐2) and reactive oxygen species (ROS) (Figure 5a‐3) when exposed to near‐infrared (NIR) light.^[^ ^9^ ^]^ The researchers then tested the LMs particles to show the same ability to absorb ultraviolet, visible, and NIR light as photosensitizers such as melanin^[^ ^11^ ^]^ and carbon‐based materials.^[^ ^79^ ^]^ Besides, previous research has also demonstrated the light‐triggered deformation ability of the LMs NPs, which was caused by the heat‐induced oxidation of Ga into an intermediate product of GaOOH (Figure 5a‐4).^[^ ^40^ ^]^ It can be found that the intrinsic causes of thermal energy and ROS generation as well as shape deformation are attributed to the light response effect of LMs NPs (fundamental phenomena: thermal, optical, and electrical) and subsequent electrochemical reactions with the ambient environment (ROS: Ga – 3e = Ga^3+^, O_2_ + e = ·O_2_, ·O_2_ + 2H_2_O + 3e = 4·OH; shape deformation:4Ga + 3O_3_+ 2H_2_O → 4GaOOH,2Ga + 4H_2_O → 2GaOOH + 3H_2_↑). Recently, the light response mechanism of LMs NPs has also been investigated.^[^ ^80^ ^]^ During light‐LMs NPs interaction, light is either absorbed (absorbed by the LMs NPs and dissipated as heat) or scattered (re‐emitted or reflected at the same/shifted frequency) when light energy is transferred to the LMs NPs. As shown in Figure 5b, LMs NPs were dissolved in ethanol, and the extinction and absorption spectra all show two dominant peaks: one broad peak centered at ≈275 nm and a second one at ≈213 nm.^[^ ^81^ ^]^ After subtracting the absorption spectrum from the extinction spectrum, it was found that the LMs NPs also strongly scatter light in the region of the 213 nm peak, while the spectrum is dominated by absorption above 220 nm (Figure 5c).^[^ ^81^ ^]^ Further optical properties of LMs NPs, the ultraviolet‐visible‐NIR spectrum, and the FTIR spectrum are shown in Figure 5d,e.^[^ ^82^ ^]^ When the frequency of incident photons matches the oscillation frequency of LMs NPs, localized surface plasmon resonances (LSPR) may occur due to the strong absorption effect on photon energy.^[^ ^83^, ^84^ ^]^ Therefore, the plasma frequency (the frequency of electron cloud oscillation) of the Ga‐based LMs lies in the energy range comparable to ultraviolet (UV) light,^[^ ^44^, ^83^ ^]^ and may also be manipulated via structure, size, and shape adjustment, realizing the shifted resonance peak from the UV region to the visible region.^[^ ^83^, ^85^ ^]^

### 2.5 Magnetic Response

As noninvasive and selective stimulus, magnetic fields (permanent and alternating magnetic fields) can be remotely applied to different forms of LMs materials to produce different phenomena.

**Figure 6:**
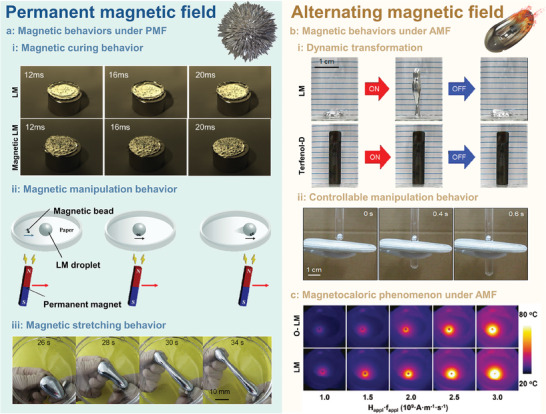
a) Magnetic behaviors of LMs‐based magnetic fluid under permanent magnetic field (PMF): i) magnetic curing behavior: high‐speed videos for the dynamic impact of striking LMs droplets (GaIn_24.5_) and magnetic LMs droplets (Ni nanoparticles loaded GaIn_24.5_) on a magnet plate. Reproduced with permission.^[^ ^90^ ^]^ Copyright 2013, Elsevier. ii) magnetic manipulation behavior: magnetic actuation for steering LMs droplet locomotion^[^ ^91^ ^]^; iii) magnetic stretching behavior: the sequential snapshots of the magnetic LMs droplets under the magnetic manipulation in the horizontal level. Reproduced with permission.^[^ ^86^ ^]^ Copyright 2019, American Chemical Society.b) Magnetic behaviors of LMs fluid under alternating magnetic field (AMF): i) dynamic transformation: morphological changes of LMs and Terfenol‐D under AMF induction Reproduced with permission.^[^ ^16^ ^]^ Copyright 2018, The Authors.ii) controllable manipulation behavior: time‐lapsed electromagnetic levitation images of an LMs droplet in water. Reproduced with permission.^[^ ^16^ ^]^ 2018, The Authors. c) The magnetocaloric phenomenon under AMF: thermal IR images of oxidized LMs and LMs with different field intensities under the AMF. Reproduced with permission. ^[^ ^17^ ^]^ Copyright 2020, John Wiley and Sons.

In general, Ga‐based LMs are non‐magnetic, so they are almost unresponsive to permanent magnetic fields (PMF). However, magnetic LMs composites that integrated magnetic and flexible characteristics can be obtained by magnetic elements doping strategy, which may exhibit magnetic curing, manipulation, and stretching behaviors under PMF. For example, in recent years, LMs have been mixed with various magnetic particles (e.g., iron, gadolinium, and nickel, as shown in **Table** **3**) to produce LMs‐based magnetic fluids via preparation methods such as mixing, surface coating, and stirring.^[^ ^86^, ^87^, ^88^ ^]^ In fact, the magnetic fluid of mercury loading with Ni and Fe nanoparticles has been studied for several years. However, the serious toxicity limited the application of mercury‐based magnetic fluid.^[^ ^89^ ^]^ As shown in **Figure** **6**a‐i, the magnetic curing behavior of LMs‐Ni magnetic fluid was investigated. When LMs fluid (without Ni particles) struck the magnet, a two‐stage process of spreading and curling was observed. While only one‐stage of the spreading process was observed when LMs‐Ni magnetic fluid struck the magnet (the curling of the LMs was restricted by magnetic attracted and neatly arranged Ni particles), indicating that LMs‐Ni magnetic fluid exhibited a magnetic curing behavior under the action of a permanent magnet.^[^ ^90^ ^]^ Besides, with magnetic properties, these magnetic LMs materials could be actuated easily and conveniently by moving a permanent magnet (Figure 6a‐ii).^[^ ^91^ ^]^ When applying two magnets in opposite directions, the magnetic LMs (LMs‐Fe) droplet could be greatly stretched as long as almost four times longer than the initial length, as shown in Figure 6a‐iii. Herein, the surface oxide formation is the key factor for the improved deformability, since the surface oxide could significantly reduce surface tension and prevent leakage of magnetic particles.

**Table 3:** Summarized magnetic LMs materials.

Type of LM	Metal added	Size of metal added	Preparation method	Coercivity (G) Negative/ positive	Saturation Magnetization (emu/g) Negative/positive	Remanent magnetization (emu/g) Negative/positive	Ref.
GaIn_24.5_	Fe	≈29–50 µm	Mix/surface coating	–	–	–	[86, 87]
GaInSn	Gd	≈nm–µm	Mix	–	–	–	[88]
GaIn_24.5_	5%Ni	≈nm	Mix and stir	−125.57/130.5	−0.8/0.8	−0.17/0.15	[90]
GaIn_24.5_	10%Ni	≈nm	Mix and stir	−138.45/138.34	−2.0/2.0	−0.34/0.34	[90]
Ga	10%Ni	≈nm	Mix and stir	−138.02/136.75	−2.3/2.3	−0.36/0.36	[90]

Under the alternating magnetic fields (AMF), LMs as conductive fluids can also be manipulated by the Lorentz force and accompanied by the magnetocaloric phenomenon. As shown in Figure 6b‐i, the LMs droplet exhibited a “stand up” behavior with a 9‐fold increase in height under AMF. This deformation was reversible when the AMF power was off, while the same behavior was not observable in Terfenol‐D due to its slight magnetostriction. In addition, upon continuous exposure to AMF, the LMs droplet floated in the water and maintained its position despite the glass tube being moving vertically (Figure 6b‐ii). Moreover, LMs also exhibited a favorable magnetocaloric effect under AMF, (the temperature increase could reach above 80 °C under AMF: frequency 100 kHz, 30 kA m^−1^), while oxidized LMs showed a lower exothermic ability (Figure 6c).^[^ ^17^ ^]^ Under AMF induction, the heating capacity of electrically conductive material is related to eddy currents generated by electromagnetic induction, which results in Joule heating.^[^ ^92^ ^]^ The mechanism involved in the magnetocaloric effect can be explained that the favorable electrical conductivity (e.g., GaIn_24.5_, EGaIn 3.4 × 10^6^ S m^−1^) of LMs enables the AMF to induce an eddy current passing through the LMs and thus produce substantial heat.^[^ ^93^ ^]^ Notably, the difference between magnetic hyperthermia conversion of conventional solid materials and LMs is the typical amplitude and frequency. Conventional materials need high‐powered AMF with a typical amplitude and frequency (Hf) on the order of 10^9^ Am^−1^ s^−1^ while that for LMs NPs could decrease to 10^7^ Am^−1^ s^−1^, thus reducing potential harmfulness to patients.^[^ ^16^ ^]^

#### 2.5.1 Microwave Response

**Figure 7:**
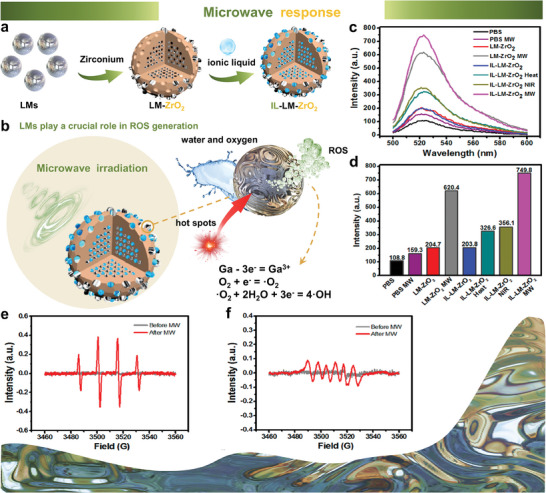
LMs NPs with microwave response. a) IL‐LM‐ZrO_2_ supernanoparticles (IL‐LM‐ZrO_2_ SNPs) were constructed by loading LMs (generation source of ROS) and ionic liquid (microwave heating enhancer) into mesoporous ZrO_2_ nanoparticles. b) The ROS generation mechanism of LMs NPs. c) Fluorescence intensity of ·OH generated in PBS and LMs NPs under different treatments. d) Maximum fluorescence intensity of ·OH generated in PBS under different treatments. Electron paramagnetic resonance (EPR) detection of LMs NPs under MW irradiation to produce e) ·OH and f) ·O_2_. Reproduced with permission.^[^ ^18^ ^]^ Copyright 2019, American Chemical Society.

Microwave (MW) energy is generally perceived to be insufficient for inducing free radical generation, which can be attributed to the low MW energy (10^−3^ eV). Very recently, EGaIn supernanoparticles have been found to generate ROS under MW irradiation, which means that LMs could serve as a MW sensitizer for revolutionizing the ROS generation techniques (**Figure** **7**a). The ROS generation mechanism has been scarcely revealed, one of the rationalities is that the portion of MW energy is concentrated into hot spots by the localized resonant coupling to point defects or weak surface bonds, which causes the generation of a free radical.^[^ ^94^ ^]^ Wu et al., revealed that LMs NPs follow a similar mechanism to generate ROS under MW irradiation (Figure 7b). The temperatures of the solution and LMs NPs rise at different rates under MW irradiation because of the internal heating by MW energy (ionic liquid as microwave heating enhancer), which induces LMs NPs to attain a higher local surface temperature than the solution. The interfacial selective heating by MW energy creates “hot spots” in the mesopores of NPs. In the hot spots, the LMs NPs use the MW energy to drive the electron transfer from Ga to the water and oxygen adsorbed in the mesopores of NPs. Thus ·OH and ·O_2_ are produced, involving the three equations: Ga – 3e = Ga^3+^, O_2_ + e = ·O_2_, ·O_2_ + 2H_2_O + 3e = 4·OH. The existence of LMs here sensitized MV energy and enhanced the generation of ROS. The researchers reported that the content of ROS produced by LMs NPs under MW irradiation was 4.7 times and 5.9 times higher than that of PBS under MW irradiation and PBS (Figure 7c,d). In addition, as shown in Figure 7e, a large amount of ·OH was generated from LMs NPs under MW irradiation. Similarly, the electron spin resonance (ESR) spectrum also manifested that superoxide anion free radical (·O_2_) was generated under MW irradiation (Figure 7f).

## 3 LMs Enhanced Energy Conversion Platforms and Applications

Because of the outstanding energy response properties (including heat response, light response, magnetic response, and microwave response), LMs are well‐suited to create unconventional energy conversion materials. While going beyond conformist concepts, LMs have been successfully applied in numerous applications that could transform the energy conversion landscape, such as heat sensitizers for enhanced heat transfer, light sensitizers for photothermal conversion, magnetocaloric conversion sensitizers for hyperthermia therapy, microwave sensitizers for dynamic therapy and medical imaging sensitizers for enhanced imaging.

### 3.1 LMs Materials as Heat Sensitizers for Heat Conversion and Transfer

#### 3.1.1 Heat Generation

Maintaining, monitoring, sensing, and analyzing skin temperature (as one of the most important physiological parameters) is of great significance, since its distribution and fluctuation can directly affect or reflect physical conditions, predict disease, and sense the external environment. When electric current flows through a resistance, the conversion of electrical energy into thermal energy is known as Joule heating. As a flexible conductive material with superior electrical conductivity, LMs materials can easily accomplish the efficient electro‐thermal conversion for functionalized flexible electronics, such as Joule heaters,^[^ ^95^ ^]^ thermo‐haptic sensation^[^ ^96^ ^]^ and human health diagnosis.^[^ ^97^ ^]^

These LMs‐based functionalized flexible electronics cannot only reduce discomfort and avoid skin inflammation owing to their permeability and comfortable tactile feeling, but also can cope with the stability challenges induced by deformations (e.g., bending and twisting) during continuous and large‐scale joint movements.^[^ ^95^, ^97^ ^]^ In the aspects of on‐skin/ wearable Joule heaters, LMs‐based flexible electronics with Peano curves and serpentine structures have been demonstrated to manifest rapid and stable electric‐thermal conversion performance, thus enabling reproducible and recoverable heat output in response to voltage variations.^[^ ^95^ ^]^ Besides, with model‐based feedback control, the temperature of the LMs‐based Joule heater can be effectively controlled to provide accurate and rapid thermo‐haptic sensation for immersive virtual reality.^[^ ^96^ ^]^ For human health diagnostics, the skin temperature can be directly accessed through the thermoelectric conversion (i.e., Seebeck effect, thermoelectric potential induced by the temperature difference between two different conductors or semiconductors).^[^ ^97^ ^]^ Meanwhile, the thermophysical parameters of biological tissues, such as thermal diffusivity, thermal conductivity and blood perfusion rate, can also be obtained by real‐time monitoring and analyzing (via bioheat transfer analysis) the temperature response of biological tissues under thermal stimulation.^[^ ^97^ ^]^

#### 3.1.2 Heat Dissipation

Since entering the electronic information age, artificial intelligence and miniaturization have been in great desire. Correspondingly, heat dissipation in these electronic products has a greater impact on their efficiency and performance. Therefore, searching for materials with a high performance of heat dissipation is of great significance for developing the electrical industry. With intrinsic superior high thermal conductivity and fluidity, room temperature LMs have been first proposed for cooling high‐performance chips by Liu and Zhou.^[^ ^8^ ^]^ Then, the prototype of LMs cooling was constructed and tested by Liu's group, and much better performance was confirmed of LMs when compared to water.^[^ ^98^ ^]^ Besides the macro LMs and their alloys, nano LMs fluid was proposed to improve the cooling capacity in 2007.^[^ ^64^ ^]^ The application of LMs cooling technology was further extended to the cooling of high‐power LEDs,^[^ ^99^ ^]^ power battery packs,^[^ ^100^ ^]^ and laser diodes.^[^ ^101^ ^]^

**Figure 8:**
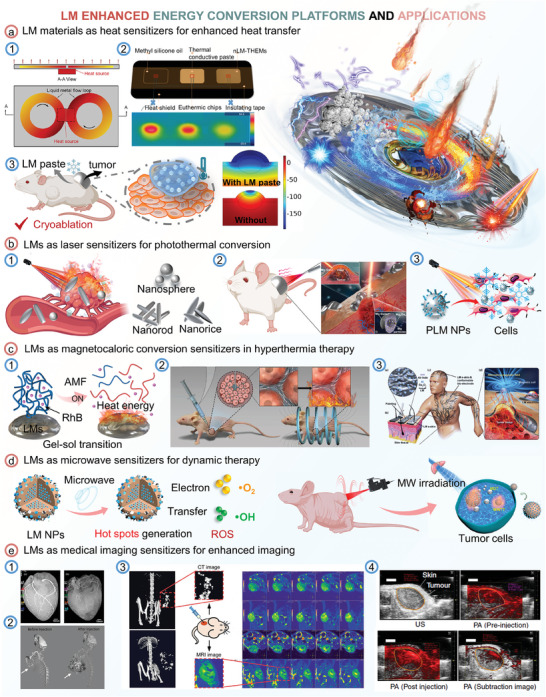
LMs enhanced energy conversion platforms and applications. a) LMs materials as heat sensitizers for enhanced heat transfer. 1) Liquid metal compact heat spreader. Reproduced with permission.^[^ ^102^ ^]^ Copyright 2018, Springer Nature. 2) Experimental device diagram of the actual cooling effect of methyl silicone oil, thermally conductive paste, and LM‐TIMs. Reproduced with permission.^[^ ^68^ ^]^ Copyright 2018, The Royal of Society Chemistry. 3) The temperature distribution of the whole tumor is more concentrated and uniform with LMs paste. Reproduced with permission.^[^ ^104^ ^]^ Copyright 2020, American Chemistyr Society. b) Applications of LMs materials as laser sensitizers used in photothermal conversion. 1) Photothermal responses of EGaIn nanodroplets with different shapes upon NIR laser irradiation. 2) Schematic illustration of Mg‐GaIn mediated heat concentration in vivo cancer PTT. Reproduced with permission.^[^ ^13^ ^]^ Copyright 2018, John Wiley and Sons. 3) Cryopreservation of HBMSCs using LMs NPs under NIR. Reproduced with permission.^[^ ^82^ ^]^ Copyright 2020, Elsevier. c) LMs materials as magnetocaloric conversion sensitizers in drug release and hyperthermia therapy. 1) Schematic illustration of gel‐sol transition of LMs‐agarose gel composite upon AMF induction. Reproduced with permission.^[^ ^16^ ^]^ Copyright 2018, The Authors. 2) AMF‐driven transformable LMs hybrid for cancer thermochemotherapy. Reproduced with permission.^[^ ^114^ ^]^ Copyright 2019, John Wiley and Sons. 3) Schematic illustration of conformable oxidized GaIn bioelectrodes on in vivo tumors under an AMF. Reproduced with permission.^[^ ^105^ ^]^ Copyright 2019, John Wiley and Sons. d) LMs as microwave sensitizers for dynamic therapy. Reproduced with permission.^[^ ^18^ ^]^ Copyright 2019, American Chemical Society. e) LMs as medical imaging sensitizers for enhanced imaging. 1) X‐ray angiogram of the heart filled with LMs Ga (left) and iohexol (right). Reproduced with permission.^[^ ^117^ ^]^ Copyright 2014, IEEE. 2) 3D renderings of in vivo CT scans before and after LMs injection. Reproduced with permission.^[^ ^114^ ^]^ Copyright 2019, John Wiley and Sons. 3) CT image and the MRI T2 image of mice after LMs injection. Reproduced with permission.^[^ ^118^ ^]^ Copyright 2020, John Wiley and Sons. 4) Ultrasound (US, gray) and photoacoustic (PA, red) images were taken through the tumor by 750 nm laser excitation. Reproduced with permission.^[^ ^9^ ^]^ Copyright 2017, Springer Nature.

As shown in **Figure** **8a‐1**, LMs circulate in a narrow channel and spread the heat from the heat source hotspot to the whole plate, then the heat could be dissipated to the ambient, which sensitized heat dissipation when compared with conventional air cooling.^[^ ^102^ ^]^ The air gap that exists between two contact surfaces which is called contact resistance often influences the heat transfer capability. Filling the gaps with thermal interface materials (TIM), which possess high thermal conductivity, can help reduce the contact resistance. Liu et al. proposed Ga‐based TIM which had thermal conductivity as high as 13.07 W/m·K. They showed that the interface resistance could be reduced by 30%−50% compared with conventional organic thermal grease.^[^ ^103^ ^]^ Fan et al. developed a nano‐LMs TIM by combining a modified polymer and LMs NPs, which realized a ≈50 × increase in thermal conductivity over the base polymer. As shown in Figure 8a‐2, the average temperature of the device with nano‐LMs TIM is the lowest compared with conventional commercial thermal conductive greases, which demonstrate the superior thermal diffusivity and thermal conductivity of nano‐LMs TIM.^[^ ^68^ ^]^ All the above results greatly demonstrated that LMs materials could act as heat sensitizers for enhanced heat dissipation.

#### 3.1.3 Heat Concentration

Thermal physical therapy is a therapeutic strategy that destroys undesired tissues by external energy intervention, so the key of thermal physical therapy is to ensure precise energy delivery. LMs materials with superior high thermal conductivity have been recently used in thermal physical therapy including cryoablation and hyperthermia to enhance heat source concentration.

As shown in Figure 8a‐3, GaIn‐Cu composite with increased thermal conductivity was introduced to enhance the heat transfer in cryoablation. Performing the same mission of heat‐conducting, the interference of high‐thermal‐conductivity LMs materials potentiates the therapeutic performance by ensuring a conformable treatment area and accurate target, as well as facilitating faster heat transfer and more uniform temperature distribution of the whole tumor area. In this case, the LMs cover the skin of the tumor‐like a quilt, which helps heat concentration for cryoablation. During cryoablation, the cryoprobe does not need to intervene in the tumor, which can not only ensure a more concentrated and uniform temperature distribution throughout the whole tumor but also reduce the pain of patients.^[^ ^104^ ^]^ In addition, soft and moldable Mg‐doped LMs for conformable tumor photothermal therapy have been investigated.^[^ ^13^ ^]^ The LMs paste with favorable formability, and high thermal conductivity could adapt to any irregular skin surface, which is conformable. On the one hand, LMs materials exert a photothermal effect, and on the other hand, heat is concentrated by targeted LMs materials on the tumor site for hyperthermia. Through fluent simulation, the temperature distribution in tumor tissues with Mg‐GaIn laser irradiation is larger and deeper than that of laser irradiation only. With Mg‐GaIn covering, the heating treatment area was expanded and concentrated, which demonstrated enhanced heat transfer. Very recently, Ga‐based functional LMs were mediated into AMF hyperthermia therapy. Wang et al. reported a kind of oxidized GaIn LMs material, which exhibited superior thermo‐/electro‐conductivity, excellent softness, and printability. The LMs material could be directly printed on the skin surface. With LMs covering the skin surface, the heat generation and thermal dissipation were balanced, and the temperature remained relatively stable without significant changes.^[^ ^105^ ^]^ With excellent thermal conductivity, LMs materials can be a potential candidate for thermal physical therapy to ensure energy delivery and enhance heat concentration.

### 3.2 LMs as Laser Sensitizers for Photothermal Conversion

In recent years, LMs materials have been surface functionalized by many researchers to study their photothermal conversion properties. In this part, we summarized and compared different types of LMs materials used in photothermal conversion (**Table** **4**). As shown in Table 4, the materials for functionalization are mostly polymers, which may accelerate LMs dispersion into micro or nanoparticles. Up to now, the laser wavelength used almost is 808 nm, which ensures effective penetration and no burns. The calculated photothermal conversion efficiency of these different LMs materials ranges from 25.3% to 53%, which shows superior strength to conventional photothermal conversion materials such as gold nanoparticles.^[^ ^21^, ^22^, ^86^ ^]^

**Table 4:** Summarized LMs materials as laser sensitizers used in light‐to‐thermal conversion.

Types of LMs	Raw material	Functionalization	Method	Size	Laser wavelength	Photothermal conversion efficiency	Ref.
Nanospheres	Ga/ EGaIn_24.5_	PEG‐HS/MNPs/PF‐127	Sonication	≈120 −220 nm	808 nm	25.3%−53%	[11, 12, 106]
Nanocapsules	EGaIn_24.5_	DSPE‐020PA, DC(8,9)PC,	Sonication and irradiation	≈150 nm	680 nm /750 nm /785 nm /808 nm	52%	[9]
Nanorods	Ga/ EGaIn_24.5_	MNPs/CTAB/PEG‐HS/HS‐HA	Sonication	≈250 nm	808 nm	28.8%−32.7%	[12]
Nanorices	EGaIn_24.5_	PEI/HA/PDMAPS	Sonication	120–175 nm	808 nm	36.7%	[11]
Microsphere	EGaIn_24.5_	Chitosan	Stir	6–80 µm	808 nm	–	[10]
Paste	EGaIn_24.5_ and Mg	Mg	Mix and stir	>50 µm	808 nm	42%	[13]
Hydrogels	EGaIn_24.5_	NIPAm, MBA, APS	Polymerization and stir	≈530 nm	808 nm	–	[110]

MNPs: Melanin nanoparticles; CTAB: cetrimonium bromide; PDMAPS: poly [2‐ (methacryloyloxy)ethyl] dimethyl‐(3‐sulfopropyl)ammonium hydroxide; MBA: N,N0‐Methylenebis(acrylamide); APS: Ammonium persulfate; DSPE‐020PA: DSPE‐PEG2000‐Amine.

Chemically functionalized LMs nanocapsules were first found to absorb NIR light to generate thermal energy by Svetlana A. et al.^[^ ^9^ ^]^ They reported that photopolymerized LMs NPs present superior photothermal conversion efficiency (52%), excellent photothermal stability, fine thermal resistances, and a wide range of laser energy absorbance for photothermal conversion. Therefore, they applied LMs nanocomplexes to cancer therapy and verified enhanced tumor elimination.^[^ ^9^ ^]^ Then, shape‐tunable LMs nanodroplets such as LMs nanospheres, nanorods, and nanorices have been synthesized and investigated for photothermal therapy. It has been found that LMs nanorices shows the best photothermal conversion efficiency (36.7%) among LMs nanospheres, nanorods, and nanorices, and LMs nanorod exhibited a higher photothermal efficiency than LMs nanospheres (Figure 8b‐1).^[^ ^11^ ^]^ The same conclusion was also obtained in Sun's research.^[^ ^12^ ^]^ Besides the organic functionalization of LMs, inorganic silica nanoshell‐based sonochemical synthesis of LMs NPs in NIR‐triggered photothermal tumor hypothermia was investigated. It demonstrated that this inorganic LMs nanosystem significantly enhanced the photothermal performance of LMs by enhanced NIR absorption, and improved photothermal stability by oxidation protection.^[^ ^106^ ^]^ Moreover, LMs NPs coated with ZrO2 with a core‐shell structure have shown ideal photothermal conversion performance and were used for antitumor therapy in the mice model.^[^ ^107^ ^]^ In addition to nanoscale LMs, mixtures of macroscopic LMs and other metal particles have also been proved to have excellent photothermal conversion properties. Conformal macroscale LMs paste was developed to realize abnormal cancer photothermal therapy (Figure 8b‐2). The soft Mg‐LMs mixtures show a superior photothermal effect with a 61.5% photothermal conversion increase concerning that of LMs itself. In addition to cancer photothermal therapy, LMs materials have been used in cryopreservation during rewarming, which requires a faster warming rate. Pluronic F127‐LMs NPs have been applied and they can act as a spatial source to significantly enhance the warming rates with NIR laser irradiation during the warming process. And as a result of its high photothermal conversion efficiency, the viability of human bone marrow‐derived mesenchymal stem cells (HBMSCs) post‐cryopreservation reached threefold higher than that obtained by the conventional warming method (Figure 8b‐3). Despite the direct use of photothermal conversion for biomedical applications, the strong light absorption properties of LMs nanoparticles have also been used to develop selective laser patterning for soft and flexible electronics.^[^ ^70^, ^108^, ^109^ ^]^ Under laser irradiation, the laser‐induced photothermal reaction can cause a high concentration of thermal stress, thereby inducing expansion, anisotropic contraction, and oxide layer rupture of the LMs nanoparticles, and finally achieving selective laser patterning.^[^ ^108^ ^]^ This efficient, convenient, elaborate, and programmed laser sintering is expected to bring progress in future soft and flexible electronics.^[^ ^70^ ^]^

### 3.3 LMs as Magnetocaloric Conversion Sensitizers in Hyperthermia Therapy

Encouraged by the promising magnetocaloric conversion efficiency as compared to conventional magnetite materials, LMs have been applied as non‐magnetic magnetocaloric conversion sensitizers in magnetic‐mediated hyperthermia (MMH). **Table** **5** summarizes magnetic LMs materials as magnetocaloric conversion sensitizers. MMH has been recently investigated due to its electromagnetic waves’ superior penetration in tissue.^[^ ^111^ ^]^ So far, various magnetocaloric conversion sensitizers such as milli‐scaled alloy thermal seeds as well as magnetic fluid composed of magnetite NPs have been used in MMH.^[^ ^112^ ^]^ However, the rather complicated and time‐consuming macroscopic implant of the thermal seeds limits their application.^[^ ^113^ ^]^ Yue et al. investigated LMs for therapeutic bioengineering under AFM, in their study, thermal‐reversible LMs hydrogel was used for controllable drug release (Figure 8c‐1).^[^ ^16^ ^]^ As depicted in Figure 8c‐1, benefiting from the magnetocaloric conversion effect of the LMs droplet, the agarose gel networks transformed from gel aggregation to disordered conformation after applying AFM, thus achieving the controllable release of rhodamine B. Then, Wang et al. developed an AMF‐driven transformable LMs hybrid for cancer thermochemotherapy (Figure 8c‐2).^[^ ^114^ ^]^ It was found that LMs exhibited a higher temperature increase than that of Fe_3_O_4_ superparamagnetic NPs under AMF due to the different heat generation mechanisms. The main cause of heat generation in Fe_3_O_4_ NPs is by Neel relaxation and Brown relaxation, while that for LMs is eddy current (AMF induced electrical current loops within conductors, according to Faraday's law of induction). In their study, LMs were PEGylation to decrease the surface tension of LMs and make LMs injectable. After the injection of LMs, mice were exposed under AMF for 20 min with and without LMs. It was demonstrated that a rapid temperature increase of tumor tissues (46 °C within 5 min) was achieved when the LMs exposure to AMF, while no temperature change was observed in mice injected without LMs and AMF exposure. The results showed excellent performance in eradicating tumors.

**Table 5:** Summarized magnetic LMs materials as magnetocaloric sensitizers.

Type of LM	Raw materials	Substance added	Preparation method	Size	Frequency of AMF	AMF conversion efficiency	Ref.
Macro particles	EGaIn_24.5_	HCl	Microinjection	≈0.3–2 mm	100 kHz	–	[17]
Micro/nano particles	EGaIn_24.5_	PEG/MS/DOX	Mix/sonication	≈µm‐nm	298 kHz	–	[114]
Paste	EGaIn_24.5_	Oxygen	Mix and stir	≈µm	100 kHz	–	[105]
Macro/micro/nano particles	EGaIn_24.5_	PEGMET	Sonication	–	245 kHz	86.3%	[16]

MS: Mesoporous silica; DOX: Doxorubicin; PEGMET: poly (ethylene glycol) methyl ether thiol;

In addition, macroscale oxidized LMs and millimeter‐scale LMs materials were applied in MMH. Wang et al. used oxidized LMs as an excellent bioelectromagnetic agent, successfully achieving wireless power transfer for multisite tumor treatment (Figure 8c‐3).^[^ ^105^ ^]^ Very recently, the eddy‐thermal heating performance of GaIn LMs was significantly improved due to the removal of its surface oxidized film and corroded holes by dilute hydrochloric acid. This work also demonstrated that the smaller particle size of Ga‐based LMPs can impair their eddy thermal effects.^[^ ^17^ ^]^ In vivo tumor ablation by using GaIn LMs is further realized in a mouse subcutaneous tumor model as well as a rat orthotopic liver tumor model. They found that LMs showed no appreciable in vivo toxicity in mice within two months, which enables its application in biomedical applications. Thus LMs could act as a promising magnetocaloric sensitizer to destroy deeply seated tumors under AMF.

### 3.4 LMs as Microwave Sensitizers for Dynamic Therapy

A high intracellular level of ROS can induce the apoptosis and autophagy of the tumor cells, which offers an opportunity for efficient and effective anticancer treatment.^[^ ^18^ ^]^ Compared with infrared light, the microwave (MW) is a superior energy source to trigger the ROS generation for tumor therapy. With MW response, LMs recently has been applied in MW dynamic therapy. Researchers designed dual‐functional super NPs by loading LMs and ionic liquid into mesoporous ZrO_2_ NPs to accomplish dynamic and thermal therapy under MW irradiation (Figure 8d).^[^ ^18^ ^]^ It has been proved that the LMs play a crucial role in MW dynamic effect (ROS generation), while the MW sensitive ionic liquid contributes to the adjuvant MW thermal effect. Under MW irradiation, the combined MW dynamic and thermal effects successfully induced the apoptosis and necrosis of tumor cells, since the favorable tumor inhibition efficacy (92.2 ± 6.8%) was realized in the subcutaneous tumor models and satisfactory survival rate (100% survival with 40% cure rate of the tumor‐bearing mice) was achieved in the orthotopic hepatocellular carcinoma mouse models. These pioneering studies demonstrated that the LMs supernanoparticles can serve as a MW sensitizer for revolutionizing the dynamic therapy.

### 3.5 LMs as Medical Imaging Sensitizers for Enhanced Imaging

The visualization of vasculature is of great significance in vascular medical image analysis.^[^ ^115^ ^]^ Angiography which can be performed by ultrasound, magnetic resonance imaging (MRI), computed tomography (CT), and X‐rays is a method to diagnose physicians and evaluate physiological conditions related to blood vessels.^[^ ^116^ ^]^ Therefore, contrast agents which are often used to improve the imaging effects are of great significance. For CT and X‐rays, the density of contrast agents plays an important role in the imaging efficacy. Recently, with inherent softness and high density, LMs have been found to realize ever‐powerful contrast X‐ray imaging as compared to conventional agents.^[^ ^117^ ^]^ As shown in Figure 8e‐1, the X‐ray imaging exhibited tremendous differences in the pig hearts infused with LMs and iohexol. The coronary network could be seen in the heart infused with LMs while only a few large vessels could be observed in the heart infused with iohexol. In addition, LMs were evaluated for the CT enhanced effect.^[^ ^10^, ^114^ ^]^ After injection of LMs, the tumor reconstructed 3D CT images show an obvious enhancement with higher CT contrast, and the result indicated that LMs enhanced CT effect could last for up to 5 days (Figure 8e‐2). Sun et al., investigated the dual‐mode imaging capability of CT and MRI in animals after intratumoral injection of LMs microparticles at the same time.^[^ ^118^ ^]^ They revealed that LMs materials could be seen under micro‐CT imaging in a tumor. A tremendous enhancement of the T2‐weight MRI effect was observed when compared to that of the control tumor in both transverse and coronal plane images of tumors (Figure 8e‐3). Moreover, it was found that mice injected with LMs nanocapsules exhibited a great enhancement of photoacoustic (PA) signal in the tumor when compared to the control tumor model (Figure 8e‐4). Researchers synthesized anti‐EGFR‐functionalized LMs nanocapsules and they found that mice injected with these LMs nanocapsules could enhance the PA signal greatly (22–102%) in the tumor at a wide wavelength range (680–970 nm).^[^ ^9^ ^]^ Therefore, we can conclude that LMs materials could be useful and effective medical imaging sensitizers (contrast agent) in MRI, X‐ray, CT and PA imaging.

## 4 Summary and Outlook

LMs featuring both fluidic and metallic properties are capable of responding to external energy stimuli, which are uniquely suited for energy conversion in many impactful applications. In this review, we have summarized and discussed representative platforms and applications of LMs covering energy conversion. Particularly, the intrinsic energy‐responsive virtues and the involved mechanism for specific energy conversion were explored, which will help deepen our insights into LMs‐based energy conversion sensitizers.

Nonetheless, understanding the interaction of LMs with external energy, identifying the intrinsic differences between multielemental and multiphase LMs, and clarifying the relationship of compositional, structural, and dimensional influences on energy‐conversion activity remain largely lacking. In order to fully exploit the potential of LMs‐based energy conversion sensitizers, the following fundamental challenges must be explored before proceeding from the research frontier to industrial applications.

1. The energetic reactions induced by LMs are complex, especially within sophisticated organisms, wherein the multi‐dimensional and multiphase interactions among energy, external environment, and LMs, the energy conversion routes as well as the energy conversion efficiency have not yet been deeply investigated. Future developments require in‐depth investigations into the underlying mechanisms of different energy conversion effects, including unique internal and interfacial interactions, phase separation behavior, and catalytic reactions, to revolutionize energy conversion technologies.
2. Considering the structural and compositional diversity of the currently developed LMs‐based energy conversion sensitizers, there is a lack of systematic and comprehensive comparative studies to assess the intrinsic energy conversion differences. It has been demonstrated that the multielemental and multiphase LMs materials (composed of different metallic elements, intermetallic compounds, or supersaturated nanoparticles) possess significant differences in physicochemical properties. Thus, substantial efforts are still needed to systematically classify and analyze the synthesis approaches, regulation methods, and energy conversion functionalities.
3. Except for a deeper investigative and theoretical understanding of LMs‐based energy conversion sensitizers, the performance‐influencing factors in specific applications, such as the effects of composition, structure, size, and surface modifications on energy conversion activity, are also of imperative concern. Although the theoretical analyses and experimental characterizations of the diverse performance‐influencing factors remain challenging, advanced characterization equipment and innovative computational models may provide a promising avenue.

With multidisciplinary research efforts, the LMs‐based energy conversion sensitizers hold great promise for epochal technological revolutions in diverse unexplored applications.

## Conflict of Interest

The authors declare no conflict of interest.

## Author Contributions

D.W. and Y.H. contributed equally to this work. All authors wrote and discussed the draft. J.L. and W.R. conceived the and supervised the research.
